# Conventional CD11c^high^ Dendritic Cells Are Important for T Cell Priming during the Initial Phase of *Plasmodium yoelii* Infection, but Are Dispensable at Later Time Points

**DOI:** 10.3389/fimmu.2017.01333

**Published:** 2017-10-16

**Authors:** Kristina Ueffing, Hanna Abberger, Astrid M. Westendorf, Kai Matuschewski, Jan Buer, Wiebke Hansen

**Affiliations:** ^1^Institute of Medical Microbiology, University Hospital Essen, University Duisburg-Essen, Essen, Germany; ^2^Molecular Parasitology, Institute of Biology, Humboldt University of Berlin, Berlin, Germany

**Keywords:** malaria, parasitic protozoan, dendritic cells, T cells, rodent

## Abstract

Dendritic cells (DCs) are highly specialized antigen-presenting cells that orchestrate adaptive immune responses to pathogens. During malaria infection pro- and anti-inflammatory T cell responses have to be tightly balanced to ensure parasite clearance without induction of severe immune pathologies. However, the precise role of CD11c^high^ DCs in this process is still discussed controversially. Here, we demonstrate that long-term depletion of conventional CD11c^high^ DCs in *Plasmodium yoelii* (*P. yoelii*)-infected diphtheria toxin (DT)-treated RosaiDTR/CD11c-cre mice interferes with the activation of CD8^+^ and CD4^+^ T cells as well as CD4^+^Foxp3^+^ regulatory T cells at early time points during infection. Moreover, systemic levels of the pro-inflammatory cytokines IFN-γ and TNF-α were decreased in *P. yoelii*-infected mice deficient for CD11c^high^ DCs compared to infected RosaiDTR controls. To further elucidate the importance of CD11c^high^ DCs during the later phase of infection, we treated RosaiDTR/CD11c-cre and control mice with DT only from day 4 of *P. yoelii* infection onward. Strikingly, this approach had no impact on the activation and IFN-γ production of CD4^+^ and CD8^+^ effector T cells. These results indicate that CD11c^high^ DCs play a crucial role in eliciting effector T cell responses during the initial phase, but are dispensable during ongoing infection with *P. yoelii*.

## Introduction

Malaria is still one of the most important infectious diseases in humans worldwide. Mostly, children under 5 years of age have a high risk to die from malaria infections and an effective vaccine is still missing. It is well established that the immune response to the malaria parasite *Plasmodium* (*P*.) has to be tightly regulated to enable efficient pathogen clearance without excessive immune activation that might result in exacerbated tissue damage and increased mortality (1, 2). During the blood-stage of infection, CD4^+^ T cells play a critical role in balancing the immune response. While effector T cells provide help to B cells and activate immunity, immunosuppressive CD4^+^Foxp3^+^ regulatory T cells (Tregs) have been shown to expand in patients infected with *P. falciparum* (3, 4) and *P. vivax* (5) and also in different malaria mouse models (6–8). Depletion of Tregs resulted in elevated T cell responses accompanied by enhanced pathogen clearance demonstrating their immunosuppressive function in the course of *Plasmodium* infection (6). However, which mechanisms and cell types are involved in activation and controlling T cell responses to keep the balance between effective parasite clearance and harmful pathological responses is still discussed controversially.

Dendritic cells (DCs) are a heterogeneous population of professional antigen-presenting cells (APC) bridging the gap between innate and adaptive immunity. They play a key role in the initiation and regulation of cell-mediated immune responses (9). After uptake of antigens, DCs process and present peptides to naïve CD4^+^ T cells in the secondary lymphoid organs *via* MHC II molecules (10) Depending on the expression of co-stimulatory molecules and the presence of cytokines distinct CD4^+^ T cell responses are elicited. Whereas upregulation of pro-inflammatory cytokines such as IL-12 contributes to effector T cell responses (11), the presence of IL-10 promotes the induction of suppressive CD4^+^ type I Tregs (11–14). Furthermore, DCs are essential for the initial activation of naïve CD8^+^ T cells by cross-presenting peptides *via* MHC I molecules (15). However, it is unclear whether DCs are unique in their ability to initiate T cell responses against *P. yoelii* infection as suggested for *P. berghei*, a *Plasmodium* strain that causes cerebral malaria (16). Moreover, it remains to be shown at which time points during infection DCs exert their function.

For analyzing the impact of conventional CD11c^high^ DCs on adaptive immunity during *Plasmodium* infection, different mouse models are available. The most widely used so-called CD11c-DTR mice harbor the simian diphtheria toxin receptor (DTR) fused to GFP under the control of the CD11c promoter (15). By injection of diphtheria toxin (DT) all DTR-positive cells, in this case conventional CD11c^high^ cells, are depleted. However, CD11c-DTR mice die within a few days upon repeated DT application, probably due to aberrant DTR expression on non-immune cells, such as epithelial cells of the gut (17, 18). Long-term DC depletion can only be achieved in radiation chimeras in which wild-type (WT) mice are reconstituted with CD11c-DTR bone marrow (17). As an alternative mouse model we made use of RosaiDTR mice, which express a loxP site-flanked STOP cassette upstream of the DTR located within the Rosa26 locus (19). By crossing these mice to CD11c-cre mice (20) the STOP cassette is irreversibly excised resulting in DTR expression specifically in CD11c^high^ cells. Double-transgenic RosaiDTR/CD11c-cre mice very well tolerate daily DT applications for at least 10 days, thus allowing for long-term depletion of CD11c^high^ DCs (21).

In this study, we aimed to dissect to which extent and at which time points conventional CD11c^high^ DCs are involved in keeping the balance between effector and inhibitory T cell function during *P. yoelii* infection. Long-term CD11c^high^ DC depletion experiments using *P. yoelii*-infected RosaiDTR/CD11c-cre mice revealed that DCs play a crucial role in T cell activation at the early phase of infection, whereas depletion of DCs during ongoing infection has no impact on effector T cell responses.

## Materials and Methods

### Mice and Parasites

RosaiDTR mice (19) (kindly provided by Ari Waisman, Mainz, Germany) and CD11c-cre mice (20) were crossed and maintained under specific pathogen-free conditions at the Animal Facility of the University Hospital Essen, Germany. Cryopreserved *Plasmodium yoelii* 17XNL (non-lethal) infected red blood cells (iRBCs) were passaged once through WT mice before being used in experimental animals. For infection 1 × 10^5^ iRBCs were injected i.v. The frequency of iRBCs (parasitemia) was determined by microscopic examination of Giemsa-stained blood films. For CD11c^high^ cell depletion, RosaiDTR/CD11c-cre mice were injected i.p. with 12 ng/g body weight of diphtheria toxin (DT; Merck, Darmstadt, Germany) starting 1 day before or at day 4 of *P. yoelii* infection and subsequently every day. Alternatively, mice were treated with DT only once 1 day prior to infection. The study was carried out in accordance with the guidelines of the German Animal Protection Law and the state authority for nature, environment and customer protection, North Rhine-Westphalia, Germany. The protocol was approved by the state authority for nature, environment and customer protection, North Rhine-Westphalia, Germany.

### Cell Isolation, Antibodies, and Flow Cytometry

The spleen is the key site for removal of parasitized red blood cells and generation of immunity (22). Therefore, splenocytes were analyzed in all experiments performed in this study. Single-cell suspensions of splenocytes were generated by rinsing spleens with erythrocyte lysis buffer and washing with PBS supplemented with 2% FCS and 2 mM EDTA. Anti-CD3, anti-CD4, anti-CD8, anti-CD11c, anti-CD49d, anti-CD335, anti-CD19, anti-B220, anti-Ly6C, anti-MHC-II, and anti-IFN-γ (all BD Biosciences, Heidelberg Germany); anti-TNF-α and anti-Foxp3 (all eBioscience, Frankfurt, Germany); anti-CD317, anti-CD69, anti-CD64, anti-FcεRI, anti-γδTCR, anti-granzyme B, and anti-CD11a (all Biolegend, London, UK) were used as fluorescein isothiocyanate, pacific blue, phycoerythrin, BD Horizon V450, allophycocyanin or peridinin-chlorophyll protein conjugates. Dead cells were identified by staining with the fixable viability dye eFlour 780 (FVD) (eBioscience, Frankfurt, Germany). Intracellular staining for Foxp3 and granzyme B was performed with the Foxp3 staining kit (eBiocience, Frankfurt, Germany) according to the manufacturer’s recommendations. Cytokine production from freshly isolated splenocytes was measured by stimulating cells with 10 ng/ml phorbol 12-myristate 13-acetate (PMA, Sigma-Aldrich, München, Germany) and 100 µg/ml ionomycin (Sigma-Aldrich, München, Germany) in the presence of 5 µg/ml Brefeldin A and 5 µg/ml Monensin (eBioscience, Frankfurt, Germany) for 4 h at 37°C, followed by intracellular staining with the respective antibody cocktail using the Foxp3 staining kit. Flow cytometric expression analyses were performed with a LSR II instrument using DIVA software (BD Biosciences, Heidelberg Germany).

### Serum Cytokine

Blood samples were collected, incubated at room temperature, and centrifuged at 6,797 × *g*. Cytokines were quantified in sera by using a Luminex Screening assay (R&D Systems, Wiesbaden, Germany) and a Luminex 200 system with Luminex IS software (Luminex Corporation, MV’s-Hertogenbosch, Netherlands) according to the manufacturer’s instructions.

### Statistical Analysis

Statistical analyses were performed with One-Way ANOVA, Student’s *t*-test for parametric and Mann–Whitney test for non-parametric distributed data with significance set at the levels of *p < 0.05, **p < 0.01, and ***p < 0.001. Normality was tested using the D’Agostino–Pearson omnibus and Kolmogorov–Smirnov test. All analyses were calculated with GraphPad Prism 5.0 Software (GraphPad Software, La Jolla, CA, USA).

## Results

### Long-term Depletion of CD11c^high^ DCs during *P. yoelii* Infection Results in Impaired T Cell Activation

**Figure 1:**
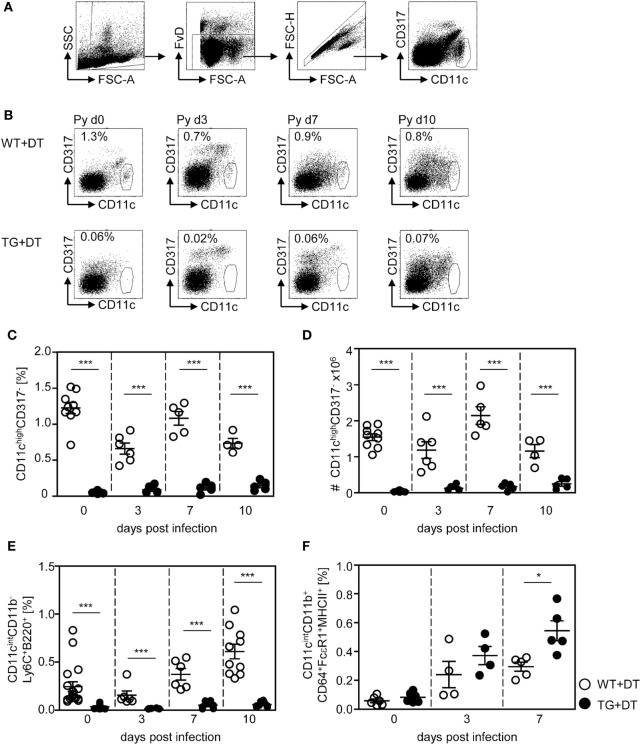
Efficient long-term depletion of CD11c^high^ dendritic cells (DCs) in diphtheria toxin (DT)-treated RosaiDTR/CD11c-cre mice. Transgenic RosaiDTR/CD11c-cre (TG) and control RosaiDTR wild-type (WT) mice were treated every day with DT (+DT) starting 1 day before infection with *Plasmodium yoelii*. At indicated time points, the percentage of splenic **(C)** CD11^high^CD137^low^ DCs was determined by flow cytometry. The gating strategy is illustrated in **(A)**, representative dot plots are shown in **(B)**. **(D)** Absolute cell numbers of CD11^high^CD137^low^ DCs were calculated and **(E)** the frequencies of CD11^int^CD11b^−^Ly6C^+^B220^+^ plasmacytoid DCs (pDCs) and **(F)** CD11c^int^CD11b^+^CD64^+^ FcεRI^+^MHCII^+^ cells were measured by flow cytometry. Data from two independent experiments with *n* = 4–9 mice per time point were summarized as mean ± SEM. Each data point represents one animal. Student’s *t*-test was used for statistical analysis with *p < 0.05 and ***p < 0.001.

To study the impact of conventional CD11c^high^ DCs on the T cell response elicited by infection with *P. yoelii*, we made use of RosaiDTR^tg/tg^/CD11c-cre^tg/wt^ mice (further referred as RosaiDTR/CD11c-cre mice). These mice express the DTR in CD11c^+^ cells due to CD11c driven cre-mediated excision of the floxed STOP cassette located upstream of the transgenic DTR. Daily treatment of double-transgenic mice with DT resulted in almost complete loss of splenic CD11c^high^CD317^−^ DCs (Figures 1B–D) as analyzed by flow cytometry (Figure 1A). As controls RosaiDTR^tg/tg^/CD11c-cre^wt/wt^ littermates (further referred to as RosaiDTR mice) were used which do not express the DTR. Injection of DT had no influence on the frequency and absolute numbers of CD11c^high^ DCs in these mice (Figures 1B–D). In addition to depletion of CD11c^high^ conventional DCs (CD11c^high^CD8^+^ and CD11c^high^CD8^−^ DCs), DT treatment also partially affects the CD11c^int^ cell compartment. We detected significant reduced frequencies of CD11c^int^CD11b^−^Ly6C^+^B220^+^ plasmacytoid DCs (pDCs) in DT-treated non-infected and infected RosaiDTR/CD11c-cre mice in contrast to RosaiDTR littermates (Figure 1E). CD11c-cre mice express the cre recombinase in more than 90% of cDCs and also in 86% pDCs (20), which might explain depletion of pDCs in RosaiDTR/CD11c-cre double-transgenic mice after DT treatment. Moreover, we detected elevated frequencies of CD11c^int^CD11b^+^CD64^+^FcεRI^+^MHCII^+^ cells in the spleen of *P. yoelli*-infected mice, which likely represent monocyte-derived DCs (moDCs) (23). Interestingly, DT treatment resulted in significant higher percentages of CD11c^int^CD11b^+^CD64^+^FcεRI^+^MHCII^+^ cells in RosaiDTR/CD11c-cre mice than in control littermates at day 7 after *P. yoelli* infection (Figure 1F). The absolute number of splenocytes increased during *P. yoelii* infection, but did not significantly differ between cDC-depleted and control mice (Figure S1A in Supplementary Material) and percentages of NK cells as well as B cells were also similar between DT-treated RosaiDTR/CD11c-cre mice and RosaiDTR littermates (Figures S1B,C in Supplementary Material).

**Figure 2:**
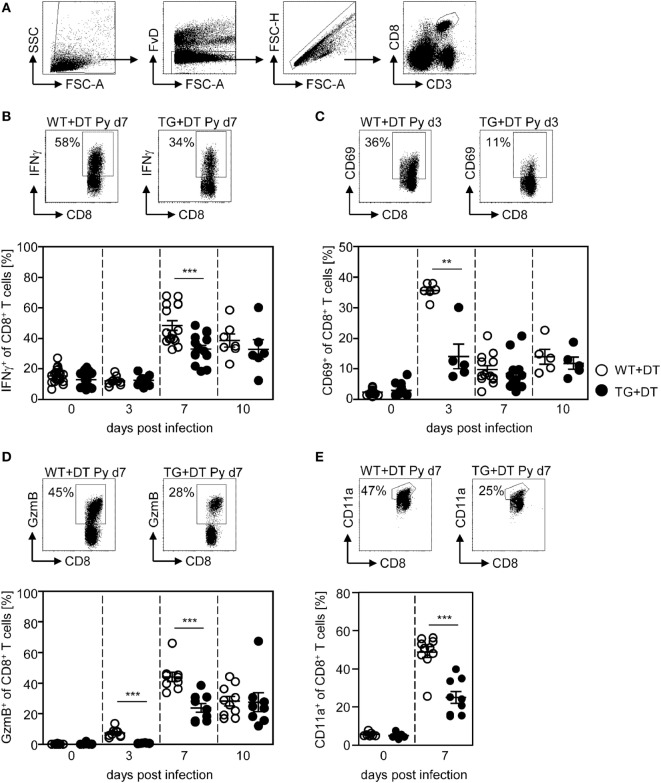
Impaired CD8^+^ T cell activation in CD11c^high^-depleted *Plasmodium yoelii*-infected mice. RosaiDTR/CD11c-cre (TG) and RosaiDTR wild-type (WT) mice were injected daily with diphtheria toxin (DT) (+DT) starting 1 day prior to *P. yoelii* infection. At days 3, 7, and 10 post infection, splenocytes were isolated and the expression of **(B)** IFN-γ, **(C)** CD69, **(D)** granzyme B (GzmB), and **(E)** CD11a was analyzed on gated CD8^+^ T cells by flow cytometry. The gating strategy is depicted in **(A)** and representative dot plots are shown in the upper panels. Results from two to five independent experiments with *n* = 5–13 mice per time point (d0; *n* = 5–25 mice) were summarized as mean ± SEM. Each data point represents one animal. Statistical analysis was performed as described in the material and methods section with **p < 0.01; ***p < 0.001.

**Figure 3:**
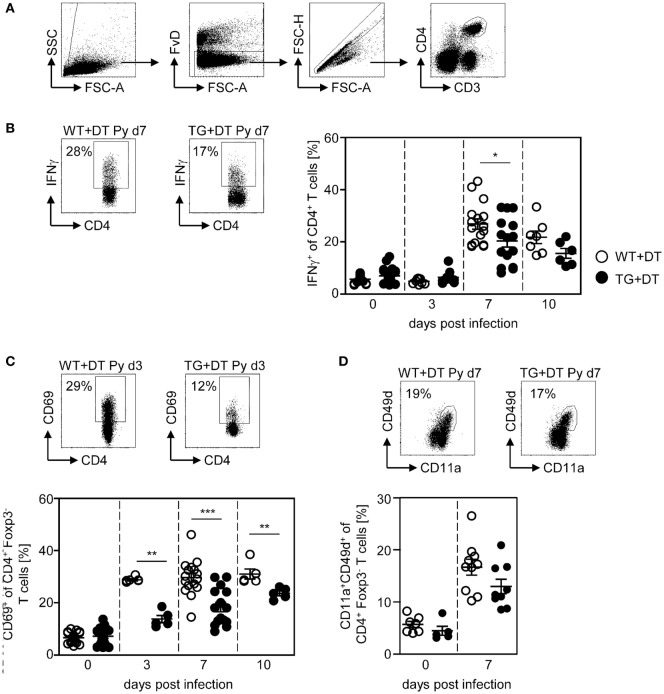
Reduced frequencies of activated CD4^+^ T cells in long-term dendritic cell (DC)-depleted *Plasmodium yoelii*-infected mice. DT-treated (+DT) RosaiDTR/CD11c-cre (TG) and RosaiDTR wild-type (WT) mice were infected with *P. yoelii* for 3, 7, and 10 days. At indicated time points, splenocytes were isolated and the frequencies of **(B)** IFN-γ-producing CD4^+^ T cells as well as **(C)** CD69-expressing and **(D)** CD11a^+^CD49^+^-expressing CD4^+^Foxp3^−^ T cells were analyzed by flow cytometry. The gating strategy is depicted in **(A)** and representative dot plots are shown in the upper panels. Results from two to five independent experiments with *n* = 5–16 mice per time point (d0; *n* = 5–25 mice) were summarized as mean ± SEM. Each data point represents one animal. Student’s *t*-test was used for statistical analysis. **p < 0.01; ***p < 0.001.

First, we investigated the effect of long-term DC deficiency on T cell activation in *P. yoelii*-infected mice. For this purpose, we treated RosaiDTR/CD11c-cre and RosaiDTR control mice with DT 1 day before infection and subsequently every day. At days 3, 7, and 10 post infection (p.i.), the phenotype of gated CD8^+^ and CD4^+^ T cells (Figures 2A and 3A) was analyzed by flow cytometry. IFN-γ production of splenic T cells was determined after re-stimulation with PMA and Ionomycin, due to the lack of identified endogenous T cell epitopes within the blood-stage *P. yoelii* parasites. As depicted in Figure 2, long-term depletion of CD11c^high^ DCs resulted in significantly reduced frequencies of IFN-γ-producing CD8^+^ T cells at day 7 p.i. (Figure 2B). Moreover, we detected lower percentages of CD8^+^ T cells expressing the early activation marker CD69 at day 3 p.i. (Figure 2C) and granzyme B (GzmB) at days 3 and 7 p.i. (Figure 2D). To investigate whether changes in the activation status of CD8^+^ T cells during long-term CD11c^high^ DC depletion is due to bystander effects or a direct result from antigen-specific stimulation, we analyzed the frequencies of CD11a-expressing CD8^+^ T cells in *P. yoelii*-infected mice. This approach was already described for the detection of antigen-specific CD8^+^ T cells during bacterial, viral, and also *Plasmodium* infections (24–26). The percentage of CD11a^+^ CD8^+^ T cells was very low in non-infected mice, while increased in *P. yoelii*-infected mice at day 7 p.i. (Figure 2E). Moreover, CD11c^high^ depletion resulted in significantly reduced frequencies of CD11a-expressing CD8^+^ T cells at day 7 after *P. yoelii* infection compared to controls (Figure 2E).

Phenotypic analysis of CD4^+^ T cells from *P. yoelii*-infected mice revealed that long-term DC deficiency interferes with IFN-γ production at day 7 p.i. (Figure 3B). In addition, the frequency of CD69-expressing CD4^+^Foxp3^−^ T cells was significantly decreased in DC-depleted mice at days 3, 7, and 10 after *P. yoelii* infection (Figure 3C). CD11a^+^CD49d^+^ staining has been shown to delineate previously activated CD4^+^ T cells from naive cells in *Plasmodium*-infected mice (27) and, therefore, represent T cells that were likely to be responding to a variety of *Plasmodium* antigens. Interestingly, long-term depletion of CD11c^high^ DCs led only to a slightly, albeit not significantly, reduced percentage of antigen-experienced CD11a^+^CD49d^+^ CD4^+^Foxp3^−^ T cells in *P. yoelii*-infected DT-treated RosaiDTR/CD11c-cre mice (Figure 3D).

**Figure 4:**
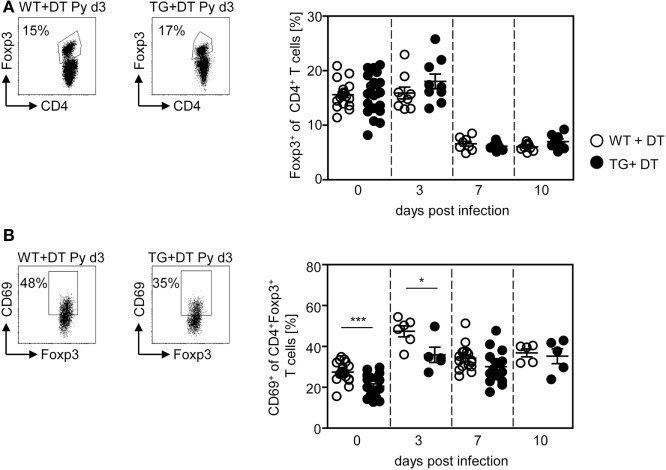
Long-term depletion of CD11c^high^ dendritic cells interferes with activation of Foxp3^+^ regulatory T cells (Tregs) during *Plasmodium yoelii* infection. RosaiDTR/CD11c-cre (TG) and RosaiDTR wild-type (WT) mice were injected daily with DT (+DT) and infected with *P. yoelii* 1 day later. Splenocytes were isolated at indicated time points and the percentages of **(A)** CD4^+^Foxp3^+^ Tregs as well as **(B)** CD69-expressing CD4^+^Foxp3^+^ Tregs were analyzed by flow cytometry. Representative dot plots are shown in the upper panels. Results from two to five independent experiments with *n* = 8–16 mice per time point (d0; *n* = 18–21 mice) were summarized as mean ± SEM. Each data point represents one animal. Statistical analysis was performed as described in the material and methods section with *p < 0.05; ***p < 0.001.

Our previous studies revealed an expansion and activation of Foxp3^+^ naturally occurring Tregs in the course of *P. yoelii* infection (6, 7). To analyze whether CD11c^high^ DCs also influence the Treg compartment, we determined the frequency and activation status of CD4^+^Foxp3^+^ Tregs in DT-treated RosaiDTR/CD11c-cre and control mice at different time points after *P. yoelii* infection by flow cytometry. Interestingly, long-term depletion of CD11c^high^ DCs did not change the percentage of Foxp3^+^ Tregs (Figure 4A), but resulted in decreased frequencies of CD69-expressing CD4^+^Foxp3^+^ Tregs in non-infected mice and at day 3 p.i. (Figure 4B). These results suggest that CD11c^high^ DCs are not only important for the activation of CD8^+^ and CD4^+^ effector T cells but also for CD4^+^Foxp3^+^ Tregs.

### Absence of CD11c^high^ DCs Interferes with Systemic Cytokine Production in *P. yoelii*-Infected Mice

**Figure 5:**
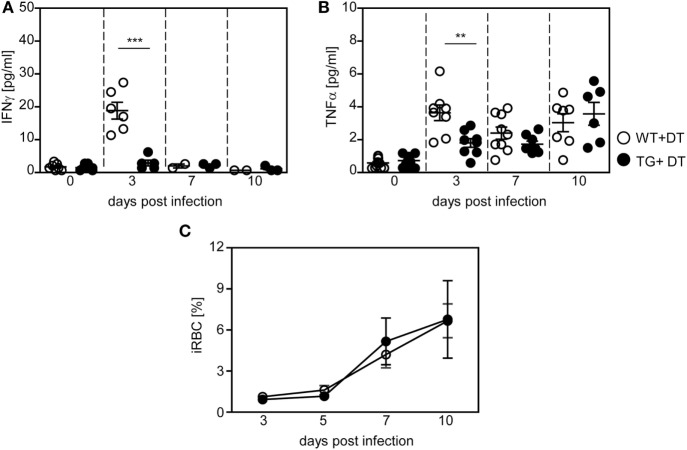
Decreased production of pro-inflammatory cytokines in long-term dendritic cell-depleted *Plasmodium yoelii-*infected mice. Diphtheria toxin (DT)-treated (+DT) RosaiDTR/CD11c-cre (TG) and RosaiDTR wild-type (WT) mice were infected with *P. yoelii* for 3, 7, and 10 days. At indicated time points, the amount of **(A)** IFN-γ and **(B)** TNF-α was determined in the serum by Luminex technology. **(C)** Parasitemia was determined at days 3, 5, 7, and 10 by Giemsa-staining. Results from two to three independent experiments with **(A,B)** *n* = 6–13 mice per time point and **(C)** *n* = 43–47 mice in total were summarized as mean ± SEM. Student’s *t*-test and One-Way ANOVA **(C)** was used for statistical analysis. *p < 0.01; ***p < 0.001.

In addition to analyzing the impact of DCs on the T cell response, we asked whether depletion of CD11c^high^ DCs modulates the systemic cytokine profile and parasite clearance. Therefore, we determined the level of pro-inflammatory cytokines in the serum of *P. yoelii*-infected DT-treated RosaiDTR/CD11c-cre and RosaiDTR controls at different time points p.i. by Luminex technology. As depicted in Figure 5, depletion of DCs resulted in significant lower production of IFN-γ (Figure 5A) and TNF-α (Figure 5B) at day 3 post *P. yoelii* infection. In addition, we detected decreased IFN-γ mRNA expression in the spleen of cDC-depleted mice in comparison to RosaiDTR control mice at day 3 p.i. (Figure S2A in Supplementary Material). To gain further insights into the cellular source of this cytokine, we determined the frequencies of IFN-γ expressing NK, NKT, and γδ T cells, because these cells have been described to produce elevated levels of IFN-γ at early time points during *Plasmodium* infection (28, 29). Well in line with these studies, we detected increased IFN-γ production by all of these cell types in *P. yoelii*-infected mice (Figures S2B–D in Supplementary Material). However, the frequencies of IFN-γ^+^CD335^+^CD3^−^ NK cells were not decreased, but the percentages of IFN-γ^+^CD335^+^CD3^+^ NKT cells and IFN-γ^+^γδTCR^+^ T cells were reduced in cDC-depleted mice compared to control littermates after infection (Figures S2B–D in Supplementary Material). These results suggest that reduced IFN-γ level in serum and spleen of cDC-depleted *P. yoelii*-infected mice are at least in part a result of lower frequencies of IFN-γ producing NKT cells and γδ T cells. In addition, we have recently shown that also CD11c^+^ DCs themselves produce elevated levels of IFN-γ and might, therefore, also contribute to higher IFN-γ production in control mice than in cDC-depleted mice during the early phase of *P. yoelii* infection (14).

Although DCs promote T cell activation and contribute to systemic production of pro-inflammatory cytokines during the early phase of *P. yoelii* infection, we detected no significant differences in parasite clearance between DT-treated RosaiDTR/CD11c-cre mice and control mice (Figure 5C). Hence, differences in T cell activation between long-term DC-depleted and control mice can directly be attributed to the presence of CD11c^high^ DCs and are not an indirect effect of lower antigen load.

### Depletion of CD11c^high^ DCs at Later Time Points during *P. yoelii* Infection Has No Impact on Effector T Cell Response and Parasite Clearance

**Figure 6:**
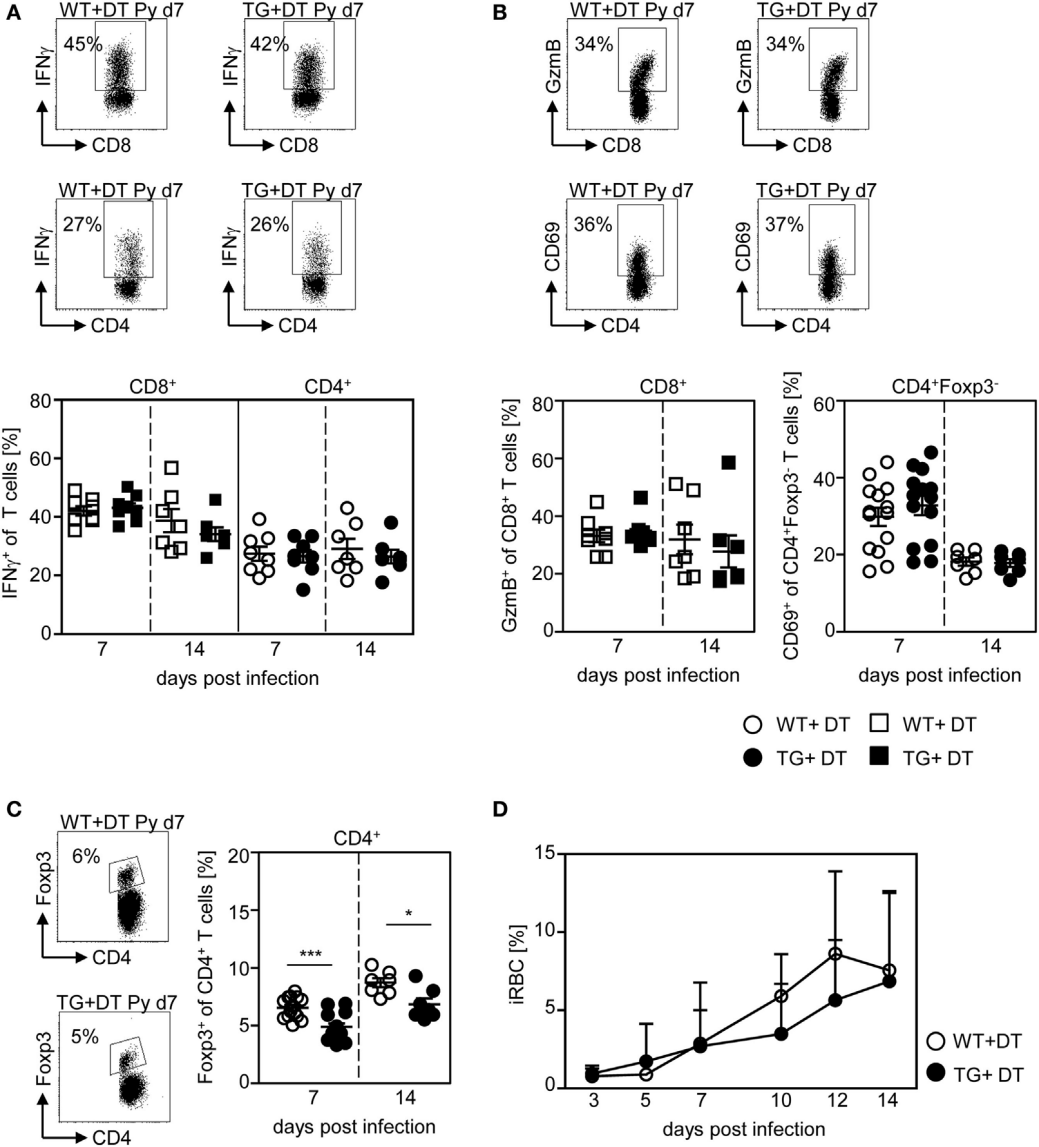
CD11c^high^ dendritic cell depletion at later time points has no impact on T cell activation and parasite burden. RosaiDTR/CD11c-cre (TG) and RosaiDTR wild-type (WT) mice were infected with *Plasmodium yoelii* and treated daily with DT (+DT) starting at day 4 post infection. At day 7 and day 14 post infection splenocytes were isolated. The percentage of **(A)** IFN-γ producing CD8^+^ and CD4^+^ T cells, **(B)** GzmB-expressing CD8^+^ T cells and CD69-expressing CD4^+^Foxp3^−^ T cells as well as **(C)** the percentage of Foxp3^+^ CD4^+^ regulatory T cells (Tregs) were determined by flow cytometry. Representative dot plots are shown in the upper panels. Results from two to four independent experiments with *n* = 7–14 mice per time point were summarized as mean ± SEM. **(D)** Parasitemia was determined at days 3, 5, 7, 10, 12, and 14 post infection by Giemsa-staining and results from two to four independent experiments with *n* = 23–24 mice in total are depicted as mean ± SEM. Statistical analysis was performed using the Student’s *t*-test and One-Way ANOVA **(D)** with *p < 0.05; ***p < 0.001.

Next, we asked whether the presence of DCs is critical at the onset of infection or whether they are able to provoke efficient T cell responses also during an established infection. Therefore, we depleted CD11c^high^ DCs from *P. yoelii*-infected RosaiDTR/CD11c-cre mice by DT treatment starting at day 4 p.i. As control RosaiDTR littermates were also treated with DT from day 4 until day 14 post *P. yoelii* infection. At day 7 and day 14 p.i., we analyzed the T cell phenotype. We did not observe any differences in the frequency of IFN-γ producing CD8^+^ T cells and CD4^+^ T cells (Figure 6A), GzmB-expressing CD8^+^ T cells and CD69-expressing CD4^+^Foxp3^−^ T cells (Figure 6B) at days 7 and 14 p.i. between mice depleted from CD11c^high^ DCs and controls. This indicates that effector T cell activation by CD11c^high^ DCs takes place during the initial phase of infection. However, DC-depletion at day 4 of *P. yoelii* infection resulted in significantly reduced frequencies of CD4^+^Foxp3^+^ Tregs (Figure 6C) and had no impact on parasite burden (Figure 6D) compared to DT-treated controls.

These results imply that long-term depletion of CD11c^high^ DCs during the entire *P. yoelii* infection modulates T cell activation and systemic production of pro-inflammatory cytokines, whereas depletion after establishment of infection has no impact on the effector T cell phenotype and parasite clearance.

### CD11c^high^ DCs Are Important for Eliciting T Cell Responses at the Onset of *P. yoelii* Infection

**Figure 7:**
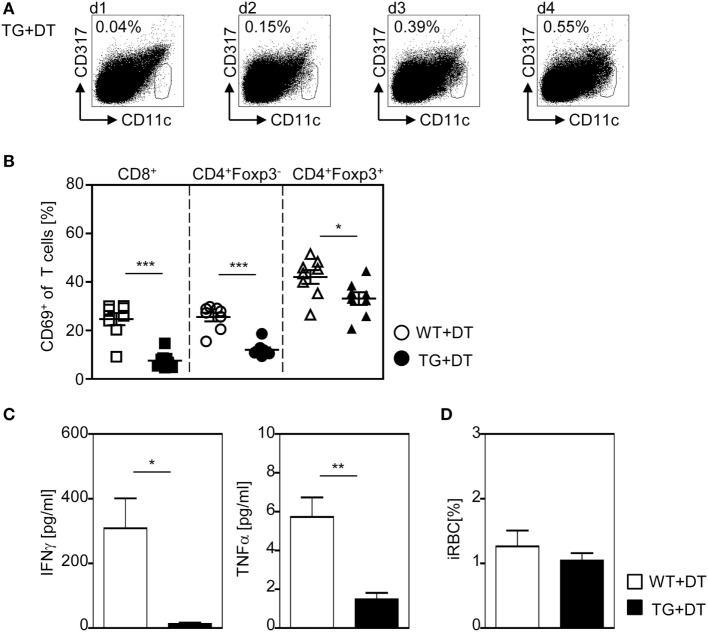
A single diphtheria toxin (DT)-injection 1 day before *Plasmodium yoelii* infection is sufficient to restrict dendritic cells (DC)-mediated T cell activation. RosaiDTR/CD11c-cre (TG) and RosaiDTR wild-type (WT) mice were treated once with DT (+DT) 1 day before infection with *P. yoelii*. **(A)** The percentage of CD11c^high^ DCs in uninfected DT-treated RosaiDTR/CD11c-cre mice was analyzed from days 1 to 4 after DT treatment in the spleen by flow cytometry. Representative dot plots are shown. At day 3 post infection **(B)**, the frequencies of CD69-expressing CD8^+^, CD4^+^Foxp3^−^ T cells, and CD4^+^Foxp3^+^ Tregs were determined by flow cytometry, **(C)** the amount of IFN-γ and TNF-α was measured in the serum by Luminex technology, and **(D)** parasitemia was determined by Giemsa-staining. Results from two independent experiments with *n* = 8 mice were summarized as mean ± SEM. Student’s *t*-test test was used for statistical analysis. *p < 0.05; **p < 0.001.

To further corroborate our finding that DC-mediated priming of T cells at early time points during infection is required to provoke efficient T cell activation, we depleted CD11c^high^ DCs by a single injection of DT to RosaiDTR/CD11c-cre mice and RosaiDTR littermates only the day before *P. yoelii* infection. This approach resulted in DC deficiency at the time point of infection lasting until 48–72 h p.i. (Figure 7A). As depicted in Figure 7B, a single injection of DT to RosaiDTR/CD11c-cre mice 1 day before *P. yoelii* infection was sufficient to significantly reduce the frequency of CD69-expressing CD8^+^ and CD4^+^Foxp3^−^ T cells as well as CD4^+^Foxp3^+^ Tregs at day 3 p.i. (Figure 7B). In addition, we detected lower serum levels of IFN-γ and TNF-α in *P. yoelii*-infected DC-depleted mice in comparison to control littermates (Figure 7C) and observed no differences in parasite burden at day 3 p.i. (Figure 7D). Strikingly, these data reflect our results from analysis of mice with long-term depletion of CD11c^high^ DCs (Figures 1–5) underlining the importance of CD11c^high^ DCs in T cell priming at early time points during *P. yoelii* infection.

## Discussion

CD11c^+^ DCs play a crucial role in the induction of antigen-specific T cell responses during a broad variety of immune responses. Thereby, modulation of their function represents a promising approach for the development of immunotherapeutic approaches and optimization of vaccine strategies. However, their precise function during *Plasmodium* infection is still discussed controversially and it is unclear at which time points during infection DCs have to execute their function. Here, we demonstrate that long-term depletion of CD11c^high^ DCs in *P. yoelii*-infected RosaiDTR/CD11c-cre mice or a single injection of DT 1 day before infection restricted T cell activation. Similar results were also described for *P. chabaudi*-infected CD11c-DTR mice, which were treated once with DT before infection (30). CD4^+^ T cells from these DC-depleted mice exhibited reduced proliferative activity associated with diminished IFN-γ production at day 4 post infection (30). Interestingly, the authors observed a significant impaired parasite clearance at late time points during infection, whereas we did not detect any differences in parasite burden between *P. yoelii*-infected DC-depleted and WT mice at least until day 10 post infection (Figure 5C). The apparent discrepancy can likely be attributed to the different experimental settings, i.e., *Plasmodium* species or more likely, the different transgenic mouse models. In CD11c-DTR mice depletion of CD11c^high^ DCs can only be achieved for a short period of time by a single DT application due to severe adverse effects of DT in these transgenic mice (17, 18). After 48–72 h newly differentiated DCs arise in parasitized CD11c-DTR mice which might exhibit functional differences and were not present in our long-term DC-depletion experimental setting. Interestingly, blood parasitemia in *P. berghei*-infected CD11c-DTR mice treated once with DT have been described to be initially lower compared to infected control mice (16). In contrast, long-term depletion of CD11c^high^ DCs in DT-treated *P. berghei*-infected bone marrow chimeric mice generated by engrafting irradiated WT recipients with bone marrow cells from CD11c-DTR mice resulted in similar parasite burden as observed in control mice (16). Well in line, we also detected comparable frequencies of infected RBCs in long-term CD11c^high^-depleted *P. yoelii*-infected mice and control mice, suggesting that CD11c^high^ DCs have not a direct effect on parasite clearance at least in the early phase of *P. yoelii* infection. However, long-term depletion of conventional CD11c^high^ DCs in DT-treated RosaiDTR/CD11c-cre mice interfered with CD69 expression and IFN-γ production of both CD4^+^ and CD8^+^ T cells, but the frequencies of antigen-experienced CD49d^+^CD11a^+^ CD4^+^ T cells were unaffected after *P. yoelii* infection. It is tempting to speculate that this might be one of the reasons for similar parasite burden in DC-depleted and control mice, since the critical role of CD4^+^ T cells during the blood-stage of infection is well established (31).

In contrast to CD4^+^ T cells, CD11c^high^ depletion resulted in significantly lower percentages of CD11a^+^ antigen-experienced CD8^+^ T cells compared to controls. Hence, CD11c^high^ DCs seem to be more important for initial cross-priming of CD8^+^ T cells than for eliciting specific CD4^+^ T cell responses during *P. yoelii* infection. However, we still detected higher frequencies of CD11a^+^CD8^+^ T cells and an elevated activation status of both CD8^+^ and CD4^+^ T cells in *P. yoelii*-infected mice than in non-infected controls. These results imply that other cells compensate for CD11c^high^ DC function. Splenic macrophages have been described to be involved in efficient T cell activation *in vivo* (32). However, the role of macrophages during *Plasmodium* infection is still unsolved. Application of clodronate, known to deplete splenic macrophages in marginal zones and red pulp (33), resulted in decreased parasite burden in *P. yoelii-*infected mice (34). By contrast, Couper and colleagues observed the opposite effect, namely a significantly elevated percentage of infected RBCs upon clodronate treatment of *P. yoelii*-infected mice (35). However, the capacity of different subsets of splenic macrophages to act as APC and their impact on T cell responses during *Plasmodium* infection has not been carefully analyzed yet.

Several studies have demonstrated that in response to infection, monocytes can be induced to differentiate into the so-called moDCs, which share many morphological and functional characteristics with cDCs, including antigen capture and presentation to T cells (36–38). Most recently, Hirako and colleagues also proposed an important role of moDCs during *P. berghei* infection. They have shown that during murine experimental malaria, splenic inflammatory monocytes differentiate into moDCs, which are CD11b^+^F4/80^+^CD11c^+^MHCII^high^DC-SIGN^high^Ly6C^+^, migrate to the brain and present there malaria antigens to CD8^+^ T cells (39). During *P. chabaudi* infection inflammatory monocytes that express low level of CD11c and low frequencies of CD11c^+^CD11b^+^F4/80^+^ cells were detected (40), which might serve as progenitors of moDCs (39). However, the use of various surface markers makes it difficult to directly compare findings on the presence and the impact of moDCs during infection. Here, we identified moDCs as CD11c^int^CD11b^+^CD64^+^FcεRI^+^MHCII^+^ cells, because CD64 (FcγRI) and FcεRI were proposed to be the best markers to discriminate between mouse cDCs and moDCs (23). Interestingly, we detected an expansion of CD11c^int^CD11b^+^CD64^+^FcεRI^+^MHCII^+^ cells in *P. yoelii*-infected mice with higher frequencies in the spleen of DT-treated RosaiDTR/CD11c-cre mice than in RosaiDTR littermates. One might speculate that this subset partially compensate for cDC deficiency in terms of T cell activation. However, further investigations are necessary to carefully dissect the role of CD11c^int^CD11b^+^CD64^+^FcεRI^+^MHCII^+^ cells during *P. yoelii* infection.

We did not observe any differences in the frequencies of Foxp3^+^ Tregs among CD4^+^ T cells during long-term depletion of DC in *P. yoelii*-infected mice, whereas starting depletion at day 4 of infection resulted in decreased percentages of Tregs. CD11c^+^ DCs have been described to be important for the regulation of Treg homeostasis (41, 42). Depletion of CD11c^+^ DCs resulted in lower percentages of Foxp3^+^ Tregs in lymphoid organs accompanied by a higher activation status of CD4^+^ T cells (41). Conversely, expanding DCs by treating naïve WT mice with recombinant Flt3 ligand led to an increase in the percentage of Foxp3^+^ Tregs (42). However, these experiments were performed with naive mice reflecting the situation under homeostatic conditions and not in the course of ongoing infection. In this study, we detected less T cell activation and production of pro-inflammatory cytokines during long-term DC-depletion associated with unchanged frequencies of Foxp3^+^ Tregs in *P. yoelii*-infected mice. By contrast, the activation status of CD4^+^ and CD8^+^ effector T cells was unaltered accompanied by reduced percentages of Foxp3^+^ Tregs within the spleen when DCs were depleted starting at day 4 p.i. Therefore, it is tempting to speculate that the elevated pro-inflammatory environment detected in *P. yoelii-*infected mice with DC-depletion at later time points in comparison to long-term DC-depletion suspends the impact of CD11c^high^ DCs on the frequency and stability of Foxp3^+^ Tregs described under homeostatic conditions. However, whether this notion can be corroborated and which mechanisms contribute to DC-dependent expansion and/or stability of Foxp3^+^ Tregs in the context of an ongoing *P. yoelii* infection have to be elucidated in further studies.

In summary, our data provide evidence that CD11c^+^ DCs are important to orchestrate T cell activation in the initial phase of *P. yoelii* infection. By contrast, depletion of DCs after the early phase of T cell priming did not change the activation status of effector T cells. Identification of cells that apparently compensate for DC deficiency and the underlining mechanisms resulting in modulation of the T cell phenotype have to be elucidated to better understand how T cell responses are orchestrated during *Plasmodium* infection as a prerequisite for the rational development of new vaccination strategies.

## Ethics Statement

The study was carried out in accordance with the guidelines of the German Animal Protection Law and the state authority for nature, environment and customer protection, North Rhine-Westphalia, Germany. The protocol was approved by the state authority for nature, environment and customer protection, North Rhine-Westphalia, Germany.

## Author Contributions

KU and HA designed and performed the experiments and analyzed data. JB, AW, and KM were involved in the data discussion and in drafting the manuscript. WH initiated, organized, and designed the study and wrote the manuscript.

## Conflict of Interest Statement

The authors declare that the research was conducted in the absence of any commercial or financial relationships that could be construed as a potential conflict of interest.

## Abbreviations

APC, antigen-presenting cell; DC, dendritic cell; iRBC, infected red blood cells; *P. yoelii, Plasmodium yoelii*; Treg, regulatory T cell.
