# Supplementary material for: Conventional CD11chigh Dendritic Cells Are Important for T Cell Priming during the Initial Phase of Plasmodium yoelii Infection, but Are Dispensable at Later Time Points
Source: Front Immunol. 2017 Oct 16;8:1333. doi: 10.3389/fimmu.2017.01333 (PMC5650681; doi:10.3389/fimmu.2017.01333)
Supplement: Supplementary file 1 [file Data_Sheet_1.PDF]

Conventional CD11c^{high} dendritic cells are important for T cell priming during the initial phase of *Plasmodium yoelii* infection, but are dispensable at later time points

Kristina Ueffing[1], Hanna Abberger[1], Astrid M. Westendorf[1], Kai Matuschewski[2], Jan Buer[1], Wiebke Hansen[1]*

[1]Institute of Medical Microbiology, University Hospital Essen, University Duisburg-Essen, Germany

[2]Molecular Parasitology, Institute of Biology, Humboldt University, Berlin, Germany

Supplemental Material and Methods

Realtime PCR

Total RNA was prepared from splenocytes using the RNeasy kit (Qiagen, Hilden, Germany) following DNase digestion (Qiagen, Hilden, Germany) and cDNA synthesis by M-MLV Reverse Transcriptase (Promega, Mannheim, Germany) and OligodT mixed with Random Hexamer primers (Invitrogen, Karlsruhe, Germany) according to the manufacturer's recommendations. Realtime PCR was performed in an ABI PRISM cycler (Life Technologies, CA, USA) using a SYBR Green PCR kit from Applied Biosystems (Life Technologies, CA, USA) and specific primers for IFN-γ (AGG AAC TGG CAA AAG GAT GGT GA and TGT TGC TGA TGG CCT GAT TGT CTT) and RPS9 (CTG GAC GAG GGC AAG ATG AAG C and TGA CGT TGG CGG ATG AGC ACA). Relative mRNA levels were determined by using included standard curves for each individual gene and further normalization to the housekeeping gene RPS9.

Supplemental Figure 1, Ueffing et al.

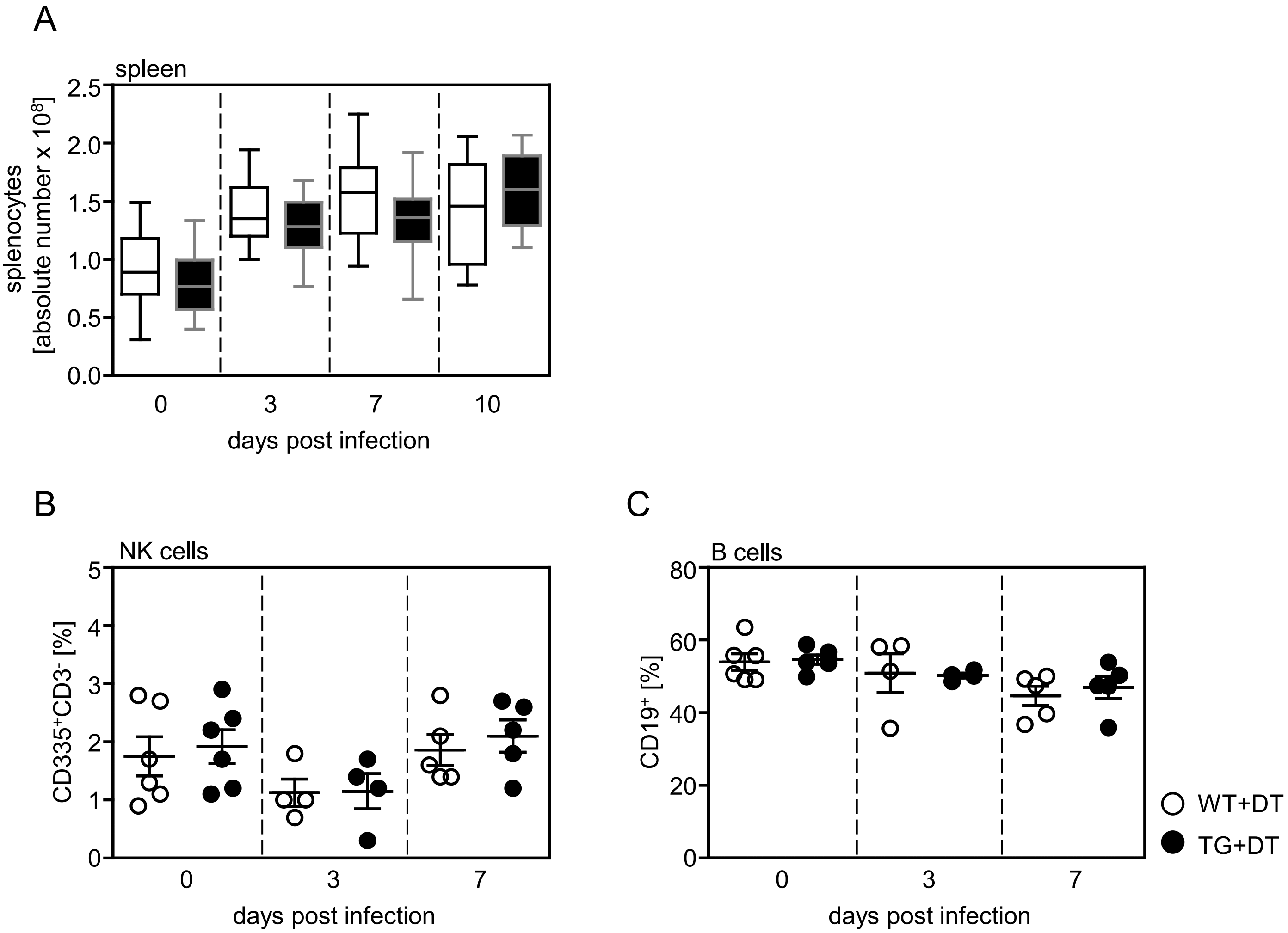

Supplemental Figure S1: CD11c^{high} cDC depletion do not affect absolute numbers of splenocytes and frequencies of NK cells and B cells within the spleen of *P. yoelii*-infected mice. RosaiDTR/CD11c-cre mice (TG) and RosaiDTR (WT) mice were treated every day with DT (+DT) starting one day before infection with *P. yoelii*. At indicated time points (A) splenocytes were isolated and counted and the frequencies of (B) $CD335^+CD3^-$ NK cells as well as (C) $CD19^+$ B cells were analyzed by flow cyotmetry. Results from 2 – 5 independent experiments with n = 4 – 16 mice ((A) d0; n = 29 – 30 mice) were summarized as mean ± SEM. Each data point represents one animal. Statistical analysis was perfomed with the Student`s t-test.

Supplemental Figure 2, Ueffing et al.

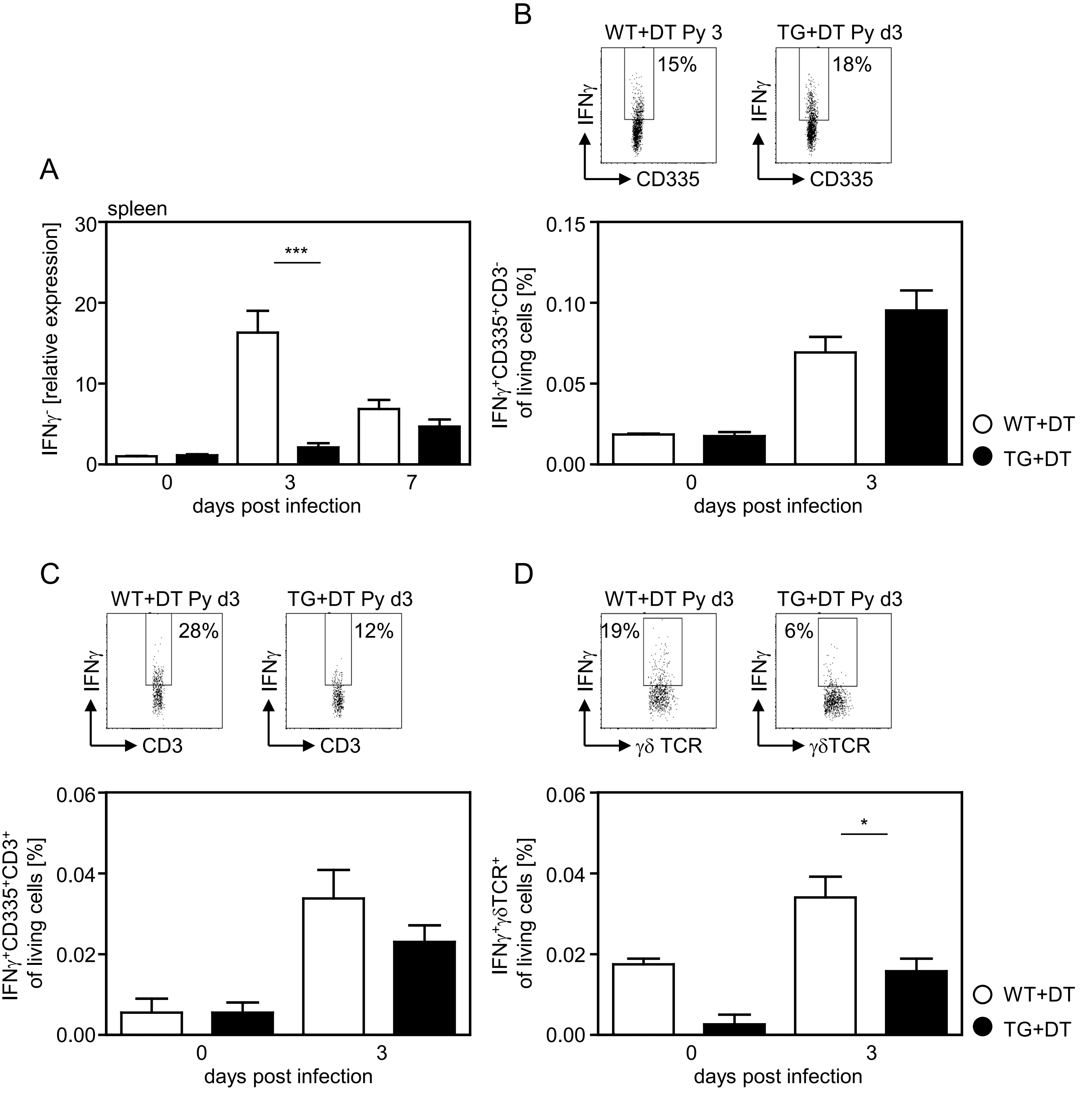

Supplemental Figure S2: Reduced IFN-γ expression in splenoctyes from cDC-depleted *P. yoelii*-infected mice. RosaiDTR/CD11c-cre mice (TG) and RosaiDTR (WT) mice were injected with DT (+DT) starting one day prior to *P. yoelii* infection. At indicated time points (A) IFN-γ expression was analyzed in spleen by Realtime PCR and frequencies of (B) IFN-γ^+CD335$^+$CD3$^-$ NK cells, (C) IFN-γ^+CD335$^+$CD3$^+$ NKT cells and (D) IFN-$\gamma^+\gamma\delta$TCR$^+$ T cells of all living cells were determined by flow cytometry. Representative dot plots showing the percentages of IFN-γ expressing cells within gated (B) CD335$^+$CD3$^-$ NK cells, (C) CD335$^+$CD3$^+$ NKT cells and (D) $\gamma\delta$TCR$^+$ T cells are depicted in the upper pannels. Results from 2 – 3 independent experiments with n = 2 – 8 mice (A, d0; n = 15 – 18 mice) were summarized as mean ± SEM. Statistical analysis was perfomed using the Student`s t-test with *p<0.05, ***p<0.001.
